# Production of site-specific antibody conjugates using metabolic glycoengineering and novel Fc glycovariants

**DOI:** 10.1016/j.jbc.2024.108005

**Published:** 2024-11-16

**Authors:** Zachary J. Bernstein, Taylor R. Gierke, Kris Dammen-Brower, Stephany Y. Tzeng, Stanley Zhu, Sabrina S. Chen, D. Scott Wilson, Jordan J. Green, Kevin J. Yarema, Jamie B. Spangler

**Affiliations:** 1 Department of Biomedical Engineering, Johns Hopkins University School of Medicine, Baltimore, Maryland, USA; 2 Translational Tissue Engineering Center, Johns Hopkins University School of Medicine, Baltimore, Maryland, USA; 3 Institute for NanoBioTechnology, Johns Hopkins University, Baltimore, Maryland, USA; 4 Department of Chemical and Biomolecular Engineering, Johns Hopkins University, Baltimore, Maryland, USA; 5 Department of Materials Science and Engineering, Johns Hopkins University, Baltimore, Maryland, USA; 6 Department of Ophthalmology, Johns Hopkins University School of Medicine, Baltimore, Maryland, USA; 7 Department of Oncology, Johns Hopkins University School of Medicine, Baltimore, Maryland, USA; 8 Bloomberg-Kimmel Institute for Cancer Immunotherapy, Sidney Kimmel Comprehensive Cancer Center, Johns Hopkins University School of Medicine, Baltimore, Maryland, USA; 9 Department of Molecular Microbiology & Immunology, Johns Hopkins University School of Public Health, Baltimore, Maryland, USA

**Keywords:** glycoconjugate, glycosylation, antibody engineering, immunoglobulin G (IgG), antibody-drug conjugate, glycoengineering, nanoparticle, Immunotherapy

## Abstract

Molecular conjugation to antibodies has emerged as a growing strategy to combine the mechanistic activities of the attached molecule with the specificity of antibodies. A variety of technologies have been applied for molecular conjugation; however, these approaches face several limitations, including disruption of antibody structure, destabilization of the antibody, and/or heterogeneous conjugation patterns. Collectively, these challenges lead to reduced yield, purity, and function of conjugated antibodies. While glycoengineering strategies have largely been applied to study protein glycosylation and manipulate cellular metabolism, these approaches also harbor great potential to enhance the production and performance of protein therapeutics. Here, we devise a novel glycoengineering workflow for the development of site-specific antibody conjugates. This approach combines metabolic glycoengineering using azido-sugar analogs with newly installed N-linked glycosylation sites in the antibody constant domain to achieve specific conjugation to the antibody *via* the introduced N-glycans. Our technique allows facile and efficient manufacturing of well-defined antibody conjugates without the need for complex or destructive chemistries. Moreover, the introduction of conjugation sites in the antibody fragment crystallizable (Fc) domain renders this approach widely applicable and target agnostic. Our platform can accommodate up to three conjugation sites in tandem, and the extent of conjugation can be tuned through the use of different sugar analogs or production in different cell lines. We demonstrated that our platform is compatible with various use-cases, including fluorescent labeling, antibody-drug conjugation, and targeted gene delivery. Overall, this study introduces a versatile and effective yet strikingly simple approach to producing antibody conjugates for research, industrial, and medical applications.

Monoclonal antibodies comprise an important and continuously growing field of biological agents with various applications in basic research and medicine, including imaging, biochemical characterization, targeted gene therapy, surface receptor modulation, and cytotoxic drug delivery (1, 2, 3, 4, 5). Many antibody applications rely on conjugating secondary agents that leverage the pinpoint specificity of antibodies to target the activities of the conjugated agents. However, current standard approaches for conjugation (such as amine or thiol chemistry) are often destructive to antibody structure and/or stability, lead to non-specific conjugation at undesired sites in the antibody, resulting in impurities that can be difficult to remove, require extensive modification of the antibody sequence, and can necessitate multi-component reactions (6, 7). As a consequence, existing conjugation strategies frequently risk adverse effects on antibody yield, stability, and purity, and potentially compromise antigen binding. Thus, there remains an unmet need for a simple, site-specific, and modular technique for antibody conjugation that circumvents the complications of harsh, non-specific, and multi-step chemistries. Such an approach would enable the rapid, high-efficiency, and homogeneous production of antibody conjugates, thereby providing chemically defined products for industrial and biomedical applications.

N-linked glycosylation is a naturally occurring co-translational modification involving the attachment of oligosaccharides to the amide nitrogen of an asparagine residue located within a specific consensus sequence (N–X–S/T, where X is any amino acid except proline) (8). This process is nearly ubiquitous for cell surface and secreted proteins, including antibodies. Metabolic glycoengineering, a technique pioneered by Werner Reutter’s group (9), takes advantage of the substrate promiscuity of biosynthetic pathways to introduce modified, non-natural sugars into endogenous glycans, allowing for chemically selective reactions (10). A pitfall for this approach, however, is that many monosaccharide analogs used in metabolic glycoengineering are hampered by poor cellular uptake. Current commercially available “high-flux”, peracetylated metabolic glycoengineering analogs utilize natural, non-specific esterase activity to enable intracellular delivery of modified monosaccharides (11, 12). Our team discovered that tri-butanoylated hexosamines, such as 1,3,4-O-Bu_3_ManNAc, which are similarly processed by intracellular esterases (12, 13), have comparatively higher flux into biosynthetic pathways, and demonstrated that these analogs can be used to supplement protein sialylation (11, 14). Additionally, these analogs can introduce non-natural chemical moieties to glycosylation pathways, including the incorporation of azido-modified sialic acid at N-linked glycosylation sites within the target protein sequence (11). Supplementation of cell cultures with these analogs thus allows for soluble proteins to incorporate chemical groups into N-glycans, enabling straightforward site-specific modification through click chemistry.

We have previously demonstrated that novel N-linked glycosylation sites can be designed into antibody variable regions and coupled with high-flux sugar analogs to improve sialylation (15). However, this approach requires bespoke engineering for each antibody sequence and also risks possible interactions with target antigen binding, especially if large drugs are attached. The fragment crystallizable (Fc) region avoids these pitfalls due to its physical separation from the antigen binding domains, and it also carries the advantage of universal applicability, independent of antibody specificity or function. The most commonly utilized Fc isotype in current clinical drugs, that of human immunoglobulin G1 (hIgG1) (1, 3), contains a canonical glycan in the Fc domain at position N297 (2). However, harnessing this glycan as a conjugation handle is difficult because this site is “buried” between the two Fc chains in a typical IgG antibody dimer. Indeed, previous attempts at using metabolic supplementation of azido-modified sugar analogs for conjugating to the N297 glycan led to low azide incorporation (16) or required concurrent disulfide reduction to incorporate thiolated analogs (17).

Here, we designed 6 novel N-linked glycosylation sites into the Fc region of hIgG1 antibodies as sites for conjugation. Click chemistry compatible azido groups were incorporated into these sites through supplementation with butanoylated metabolic glycoengineering analogs to create new handles for direct antibody conjugation. We demonstrated the utility of our modular platform in multiple applications, including fluorophore coupling, antibody-drug conjugate (ADC) design, and nanoparticle-based targeted gene delivery. By coupling protein engineering with metabolic glycoengineering, we have developed a simple, versatile, and site-specific workflow to produce chemically functionalized antibodies without the need for extensive or harsh modifications.

## Results

### Design of Fc glycovariants

**Figure 1:**
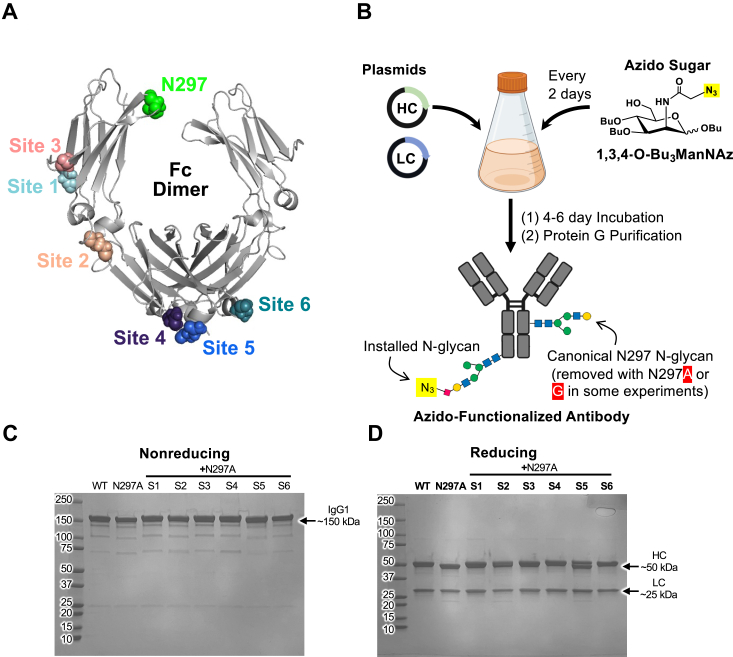
**Fc glycovariant design and manufacturing process.***A*, crystallographic structure of human immunoglobulin G1 (IgG1) fragment crystallizable (Fc) domain (PDB ID:5JII), with glycovariant sites and canonical N297 glycan site indicated. *B*, process of Fc glycovariant manufacturing through supplementation with 1,3,4-O-Bu3ManNAz, an azido-functionalized ManNAc analog. *C* and *D*, Nonreducing (*C*) and reducing (*D*) SDS-PAGE analysis of the F5111 antibody in wild type (WT), N297A, and engineered Fc glycovariant formats. HC, heavy chain; LC, light chain.

**Figure 2:**
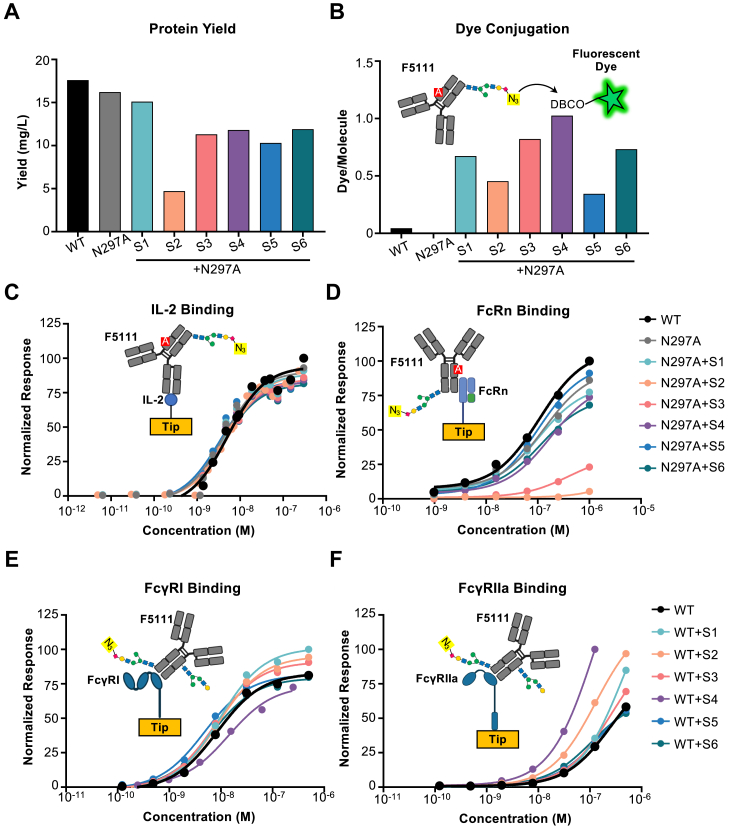
**Fc glycovariants express robustly and incorporate azides to allow fluorescent labeling while retaining antigen and Fc receptor binding.***A*, yield of antibodies from human embryonic kidney (HEK) 293F cells following purification with protein G. *B*, average number of dye molecules per antibody molecule, determined by dye/protein ratio of azide-functionalized wild type (WT) F5111 antibody and glycovariants thereof labeled with dibenzocyclooctyne (DBCO)-linked fluorescent dye, as measured by UV/Vis spectroscopy. *C*, biolayer interferometry studies of the equilibrium binding between immobilized IL-2 and soluble F5111 glycovariants. *D*, biolayer interferometry studies of the equilibrium binding between immobilized FcRn and soluble F5111 glycovariants. *E* and *F*, biolayer interferometry studies of the equilibrium binding between immobilized FcγRI (*E*) or FcγRIIa (*F*) and soluble F5111 glycovariants with re-introduced N297 glycosylation site.

To design novel N-linked glycans in the Fc region, we adopted a computational and structurally guided approach. A sliding window along the human IgG1 heavy chain (HC) constant 2 (CH2) and CH3 sequences was used to screen all potential N-X-T/S sequons. Sites were first screened for probability of glycosylation to select those with a likelihood of N-glycosylation > 0.6, as determined by the NetNGlyc server (18). Amongst these sites, six were chosen based on sequence and structural criteria, prioritizing low sequence modification as well as high solvent exposure and flexible loop secondary structure based on PyRosetta modeling (19) from the hIgG1 Fc structure (PDB ID: 5JII) (Figs. 1*A* and S1, and Table S1) (20). The six selected N-glycosylation sites were separately installed into the heavy chain of a representative hIgG1 antibody, the anti-interleukin-2 (IL-2) antibody F5111 (21). Each of the resulting F5111 glycovariants also contained the N297A substitution to allow for analysis of the engineered glycosylation sites without confounding effects from the canonical N297 Fc glycan. Each construct was then expressed recombinantly in human embryonic kidney (HEK) 293F cells, with the azido-modified ManNAc analog 1,3,4-O-Bu_3_ManNAz (100 μM) supplemented every 2 days (Fig. 1*B*). The wild-type (WT) F5111 antibody, the aglycosylated N297A mutant, and all six glycovariants (denoted S1-S6) expressed as intact IgGs with minimal impurities, as determined by SDS-PAGE analysis (Fig. 1*C*). Glycosylation of all six glycovariants was detected by molecular weight shifts relative to the aglycosylated N297A WT antibody (Fig. 1*D*). Interestingly, construct S5 showed 2 distinct heavy chain species, likely indicating a mixture of glycosylated and aglycosylated heavy chains. Each construct was expressed recombinantly, and yields were similar to the WT antibody with the exception of S2, which exhibited lower expression (Fig. 2*A*). Overall, our installed amino acid substitutions successfully introduced glycosylation with minimal effects on antibody purity or yield.

### Engineered glycovariant antibodies can be coupled to azide reactive dye and retain functional properties

Each of the expressed F5111 glycovariants was subjected to labeling with azide reactive dibenzocyclooctyne (DBCO) dye derivatives, and the average number of azides per molecule was estimated by the dye/protein ratio. All six of the glycovariant antibody sites exhibited dye incorporation, demonstrating that our novel glycan sites incorporated azido-modified sialic acids due to 1,3,4-O-Bu_3_ManNAz supplementation and, furthermore, that these groups were sterically available for click chemistry conjugation reactions (Fig. 2*B*). Importantly, all 6 novel sites (which lacked the N297 glycan) exhibited superior labeling compared to the WT antibody (which contained the N297 glycan). Labeling of the N297 glycan was low (Fig. 2*B*), consistent with previous reports (16, 17). This may be due to the inaccessibility of the buried site between the two heavy chains in the hIgG1 structure and/or low sialylation. Divergence in labeling between the different F5111 glycovariants could be due to site-to-site variation in glycoforms, which has been observed in the context of various other proteins (22).

To demonstrate that antibody-antigen binding properties were not disturbed by the Fc mutations used to install N-glycans, biolayer interferometry studies were performed against immobilized IL-2, the target protein of the F5111 antibody. As expected, the IL-2 binding properties of the F5111 glycovariants were equivalent to those of the WT antibody, with the equilibrium dissociation constant (K_D_) of each glycovariant showing a similar value of ≈4 nM (Fig. 2*C*, Table S2).

An important consideration for antibody pharmacokinetic properties is their ability to bind the neonatal Fc receptor (FcRn) through interactions with the Fc domain, which prolongs half-life by rescuing the antibody from lysosomal degradation following cellular uptake (23). Biolayer interferometry-based binding studies against human FcRn demonstrated that four of the six F5111 glycovariants exhibited similar interaction affinity compared to the WT antibody (Fig. 2*D*, Table S3). Variants S2 and S3 showed reduced affinity for FcRn, suggesting they could exhibit faster clearance. For cases wherein Fc effector function is required through interaction with Fcγ receptors (FcγR) on immune cells, the glycovariants engineered here would not be appropriate because the N297 glycan is necessary for the Fc domain to bind these receptors (24, 25). To this end, we reincorporated the N297 glycan site into each of the F5111 glycovariants and expressed the corresponding proteins in the presence of 100 μM 1,3,4-O-Bu_3_ManNAz. Overall, biolayer interferometry studies revealed that most Fc glycovariants that contained the canonical N297 glycan site showed similar binding responses to FcγRI and FcγRIIa as compared to the WT antibody (Fig. 2, *E* and *F*, Table S4). Collectively, these results demonstrate the successful incorporation of conjugation-accessible azido-modified sugar analogs into the antibody Fc domain without disruption to the target binding or Fc receptor binding functions of the modified antibodies.

### Fc glycoengineering approach is effective across antibodies of distinct specificities

**Figure 3:**
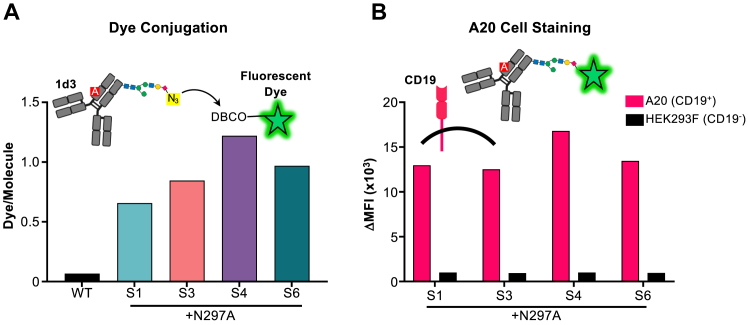
**Fc glycosylation sites can be grafted onto antibodies of distinct specificity for fluorescent labeling.***A*, average number of dye molecules per antibody molecule, determined by dye/protein ratio of azide-functionalized wild type (WT) 1d3 antibody and glycovariants thereof labeled with DBCO-linked fluorescent dyes, as measured by UV/Vis spectroscopy. *B*, mouse CD19^+^ A20 B cell staining of fluorescently labeled 1d3 glycovariant antibodies, as detected *via* flow cytometry. CD19^-^ (HEK 293F) cells were used as a negative control.

To illustrate the versatility of our Fc glycoengineering approach, we examined whether the newly installed N-glycan sites could be incorporated into an antibody with a different specificity by grafting the variable domain of the anti-mouse CD19 (mCD19) antibody clone 1d3 (26) into the hIgG1 backbone. Due to the low yield of the S2 mutant (Fig. 2*A*) and the incomplete glycosylation of the S5 mutant (Fig. 1*D*) in the context of the F5111 glycovariants, we did not include these mutants in further analyses. We grafted the S1, S3, S4, and S6 Fc glycovariant sites onto a hIgG1 DNA backbone (with the N297A mutation) containing the rat HC variable region of 1d3 and co-transfected these DNA plasmids with a plasmid encoding the rat light chain (LC) variable domain and the human kappa constant domain into HEK 293F cells, supplementing with 100 μM 1,3,4-O-Bu_3_ManNAz every 2 days. The harvested 1d3 glycovariants exhibited high purity and showed similar heavy chain molecular weights to the WT 1d3 antibody, which contains the N297 glycan (Fig. S2), suggesting successful glycosylation of the variants. DBCO dye derivative coupling studies showed similar levels of dye conjugation to 1d3 glycovariants compared to those observed for the F5111 glycovariants (Fig. 3*A*). Furthermore, the 1d3 glycovariants specifically stained the mCD19-expressing A20 mouse B cell line (Fig. 3*B*), demonstrating that, as in the case of the F5111 antibody, glycoengineering of the 1d3 antibody did not interfere with antigen recognition. Moreover, cell binding further indicated that dye conjugation also did not disrupt antigen binding, highlighting the general applicability of our platform.

### Glycan incorporation can be tuned through the use of various analogs, production in alternative cell lines, and introduction of multiple glycosylation sites

**Figure 4:**
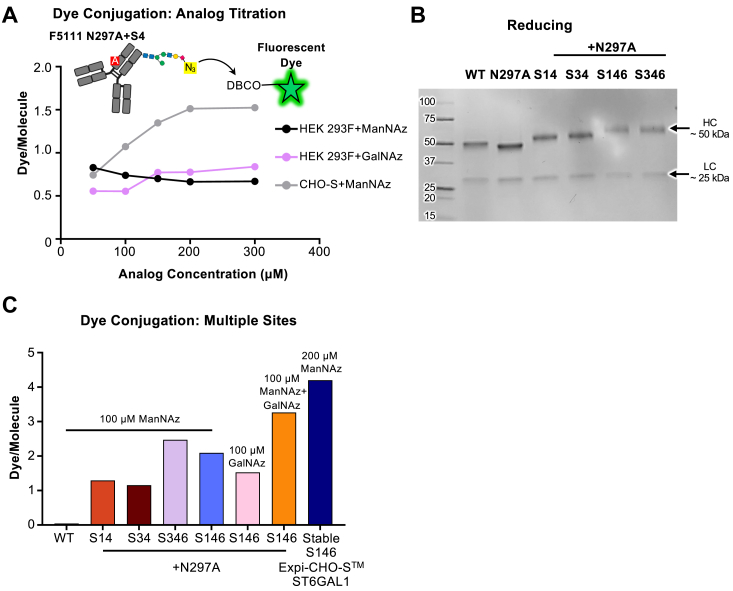
**Azide incorporation in Fc glycovariants can be optimized through use of different analogs, production in alternative cell lines, or incorporation of multiple glycosylation sites.***A*, average number of dye molecules per antibody molecule, determined by dye/protein ratio of azide-functionalized and DBCO-linked fluorescent dye-labeled S4 F5111 glycovariant antibody produced with varying amounts of 1,3,4-O-Bu_3_ManNAz (ManNAz) or Bu_4_GalNAz (GalNAz) in HEK 293F or Chinese hamster ovary (CHO)-S cells, as measured by UV/Vis spectroscopy. *B*, reducing SDS-PAGE analysis of wild-type (WT) F5111 antibody and glycovariants thereof, including double and triple glycomutant antibodies. *C*, the average number of dye molecules per antibody molecule upon expression of antibody glycovariants with supplemented azide-functionalized analogs. Either WT F5111 antibody and double or triple mutant glycovariants thereof transiently expressed in HEK 293F cells or the S146 trastuzumab glycovariant stably expressed in ExpiCHO cells in the presence of the sialyltransferase ST6GAL1 were labeled with DBCO-linked fluorescent dye, and dye/protein ratio was measured by UV/Vis spectroscopy. HC, heavy chain; LC, light chain.

To optimize azido-modified sugar analog incorporation, we titrated the concentration of analog during expression of the S4 F5111 glycovariant. We noted that in HEK 293F cells, the 100 μM concentration previously implemented for the 1,3,4-O-Bu_3_ManNAz analog led to maximal analog incorporation (Fig. 4*A*), and further improvement was not observed with increasing analog concentrations. We also tested the incorporation of a Bu_4_GalNAz analog into the S4 F5111 glycovariant, and we found that a similar number of azide sites were introduced, reaching saturation at an analog concentration of 150 μM (Fig. 4*A*). As maximum incorporation for both analogs was below 1 dye molecule per antibody, we hypothesized that the non-natural sugar analog was likely outcompeted by natural sialic acids being installed into the antibody glycans by the HEK 293F cells. To explore this possibility, we expressed the S4 F5111 glycovariant in the presence of titrated amounts of 1,3,4-O-Bu_3_ManNAz in Chinese hamster ovary (CHO)-S cells, which show lower levels of natural sialylation compared to HEK 293F cells (27). We found that glycovariants produced in CHO-S cells reached saturating levels of labeling at an analog concentration of 200 μM. Also, consistent with our hypothesis, we observed that antibody production in CHO-S cells *versus* HEK 293F cells leads to the incorporation ∼1.5-fold more dye equivalents per protein (Fig. 4*A*), likely due to the lower endogenous sialylation of CHO-S cells.

To further increase the number of drug conjugation sites, we designed double and triple mutants of the F5111 antibody (containing the N297A substitution) that combined our validated engineered sites for N-glycan installation. Double variants (S14 and S34) and triple variants (S146 and S346) exhibited notably higher molecular weight heavy chains compared to the WT (N297-containing) and N297A mutated F5111 antibodies, consistent with the incorporation of additional glycans (Fig. 4*B*). We first examined whether the incorporation of multiple glycans into the Fc region would alter various binding properties of the antibody. Encouragingly, we noted that antigen binding was not impacted, as double and triple F5111 glycovariants showed identical binding to human IL-2 compared to the parent F5111 antibody (Fig. S3*A*, Table S2). Variants containing the S3 site (either S34 or S346) showed reduced affinity and level of binding towards FcRn, whereas the S14 and S146 variants showed equivalent affinity but reduced levels of binding (Fig. S3*B*, Table S3). To evaluate FcγR binding, we reincorporated the N297 glycan into the double and triple F5111 glycovariants and expressed the corresponding proteins (Fig. S3*C*). All double and triple mutants showed a similar affinity for FcγRI with a slightly attenuated level of binding (Fig. S3*D*, Table S4). Binding of all double and triple mutants to FcγRIIa was greatly reduced, but still detectable (Fig. S3*E*). Altogether, these studies established that our glycovariants did not impact antigen binding and that glycovariants can be chosen to have minimal effect on binding to various FcRs. Double variants showed a minor increase in the extent of conjugation compared to the single variants (>1 dye molecule per antibody), whereas the triple variants demonstrated incorporation of 2 or more dye molecules per antibody (Fig. 4*C*). To increase the incorporation of azides into the engineered glycans, we chose to express the triple site S146 in the presence of both the 1,3,4-O-Bu_3_ManNAz and Bu_4_GalNAz analogs, which led to ∼3 dye molecules per antibody. To further demonstrate the versatility and developability of our conjugation platform, we generated a stable pool of (CHO-S-derived) ExpiCHO cells overexpressing the FDA-approved anti-human epidermal growth factor receptor (HER2) antibody trastuzumab containing the triple variant S146 Fc domain and an N297G mutation along with the sialyltransferase ST6GAL1, which has previously been shown to enhance sialylation when coupled with ManNAc analog supplementation (28). In this case, the glycosylated substitution N297G (as opposed to N297A) was used, as it has shown greater promise in terms of downstream developability (29). This stable pool incorporated ∼4.2 dye molecules per antibody when supplemented with 1,3,4-O-Bu_3_ManNAz (Fig. 4*C*), reinforcing that CHO-S cell lines are compatible and potentially superior expression lines for our glycoengineering platform. Overall, these studies demonstrated that the expression system, sugar analogs, and number of engineered sites can all be tuned to manipulate the extent of antibody conjugation.

### Engineered Fc glycovariant antibodies enable the design of therapeutically effective antibody-drug conjugates

**Figure 5:**
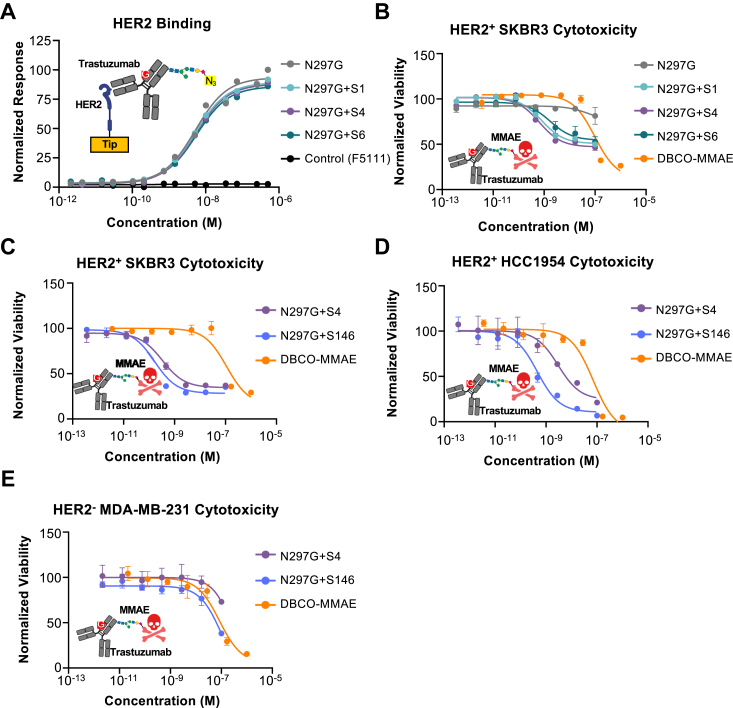
**Fc glycovariants demonstrate potent tumor cell killing upon formulation as antibody-drug conjugates.***A*, biolayer interferometry studies of the equilibrium binding between immobilized human epidermal growth factor receptor 2 (HER2) and soluble trastuzumab glycovariant antibodies. *B*, cytotoxicity of single mutant trastuzumab glycovariant antibody-drug conjugates (ADCs) against HER2-expressing SKBR3 human breast cancer cells. DBCO-linked monomethyl auristatin E (MMAE) was used as the drug payload only control. *C*–*E*, cytotoxicity of a single and triple mutant trastuzumab glycovariant ADCs against HER2^+^ SKBR3 (*C*), HER2^+^ HCC1954 (*D*), or HER2^-^ MDA-MB-231 (*E*) human breast cancer cells. DBCO-linked MMAE was used as the drug payload only control. Error bars represent standard deviation (n = 4).

Having established the efficacy of our approach in facilitating fluorescent labeling for applications in research and discovery, we sought to demonstrate the potential for using our strategy to develop novel biotherapeutics. We selected the clinical anti-HER2 antibody trastuzumab as a model therapeutic due to its current use in multiple antibody-drug conjugates (1, 30, 31). Due to their minimal effect on FcRn binding, sites S1, S4, and S6 were grafted onto the hIgG1 trastuzumab HC, and the N297G substitution was used as opposed to N297A due to its more favorable developability properties (29). Each trastuzumab glycovariant was expressed from HEK-derived Expi293F cells in the presence of 100 μM 1,3,4-O-Bu_3_ManNAz, added every 2 days. All of the resulting glycovariants exhibited high purity by SDS-PAGE and analytical high-performance liquid chromatography (HPLC), and HC molecular weight shifts were observed compared to the N297G glycomutant, consistent with glycan incorporation (Figs. S4, *A* and *B*). Moreover, as observed with F5111 and 1d3, glycomutants for trastuzumab showed an identical binding affinity for HER2 compared to the parent antibody (Fig. 5*A*, Table S2). These glycovariants exhibited similar DBCO dye derivative conjugation compared to the single mutant F5111 and 1d3 glycovariants (Fig. S4*C*).

ADCs, which link a disease-targeted antibody to a cytotoxic drug, represent a swiftly growing class of therapeutics, with 13 FDA-approved molecules (including eight in the last 5 years) and over 100 others in various stages of clinical development (1, 30, 31). To demonstrate that our glycoengineering platform can be used to generate ADCs, we linked our trastuzumab glycovariants to the tubulin inhibitor monomethyl auristatin E (MMAE), which is the most commonly employed cytotoxic payload in clinically approved ADCs (30, 31). To this end, the S1, S4, and S6 trastuzumab glycovariants were conjugated to DBCO-PEG4-Val-Cit-MMAE (DBCO-MMAE), and the cytotoxic activity of the resulting MMAE-linked anti-HER2 ADCs was evaluated against various human breast cancer cell lines. Each of the trastuzumab glycovariant ADCs exhibited cytotoxicity against the HER2-high cell line SKBR3 with potent (<2 nM) half maximal inhibitory concentration (IC_50_) (Fig. 5*B*, Table S5). The unconjugated N297G trastuzumab glycomutant did not induce cytotoxicity, and DBCO-MMAE induced significantly weaker cytotoxicity compared to the ADCs (IC_50_ ˜ 100 nM) (Fig. 5*B*, Table S5), corroborating the targeted killing achieved by the glycovariant ADCs. For the HER2-expressing cell line MDA-MB-453, the unconjugated N297G trastuzumab glycomutant led to very potent cytotoxicity (IC_50_ ˜ 230 PM), as previously reported (32), and our engineered trastuzumab glycovariants ADCs did not further potentiate killing (Fig. S5*A*, Table S5). The HER2^+^ cell line HCC1954, which has been shown to be less responsive than SKBR3 to MMAE-conjugated ADCs (33), showed reduced sensitivity to ADC-mediated killing. Notably, in this context, the S4 trastuzumab glycovariant ADC induced more cytotoxicity than the S1 and S6 trastuzumab glycovariant ADCs (Fig. S5*B*, Table S5), likely due to its higher degree of conjugation (Fig. S4*C*). Neither the unconjugated N297G trastuzumab glycomutant nor any of our engineered trastuzumab glycovariant ADCs induced cytotoxicity in the HER2^-^ MDA-MB-231 cell line (Fig. S5*C*), again confirming antigen-dependent cell killing. To augment payload conjugation and thereby enhance the tumor cell killing activity of our engineered ADCs, we generated an ADC using one of our triple mutant glycovariants. The resulting S146 trastuzumab glycovariant expressed with minimal impurity (Fig. S4, *A* and *B*), exhibited similar dye conjugation (Fig. S4*C*) compared to the S146 F5111 glycovariant (Fig. 4*C*), and retained antigen binding to HER2 (Fig. S4*D*, Table S2). The S146 trastuzumab glycovariant was formulated as an ADC *via* DBCO-MMAE conjugation, and the resulting molecule induced potent cell killing (IC_50_ < 450 PM, Table S5) of both SKBR3 (Fig. 5*C*) and HCC1954 (Fig. 5*D*) cells, with minimal cytotoxicity towards HER2^-^ MDA-MB-231 cells (Fig. 5*E*). Whereas the triple mutant S146 trastuzumab glycovariant ADC showed only incremental enhancement compared to the already high potency killing by the single mutant S4 trastuzumab glycovariant ADC (Fig. 5*C*), the S146 trastuzumab glycovariant ADC was nearly 5 times more potent than the S4 trastuzumab glycovariant ADC on HCC1954 cells (Fig. 5*D*), illustrating the advantage for increased drug conjugation levels in the context of cell lines that are less sensitive to cytotoxic agents. Altogether, ADC design efforts demonstrate that our platform can be used to produce effective and specific molecules that show tumor cell killing potency on par with current state-of-the-art ADCs (6, 7, 31, 34).

### Engineered Fc glycovariant antibody conjugation to biomaterials enables targeted gene delivery

**Figure 6:**
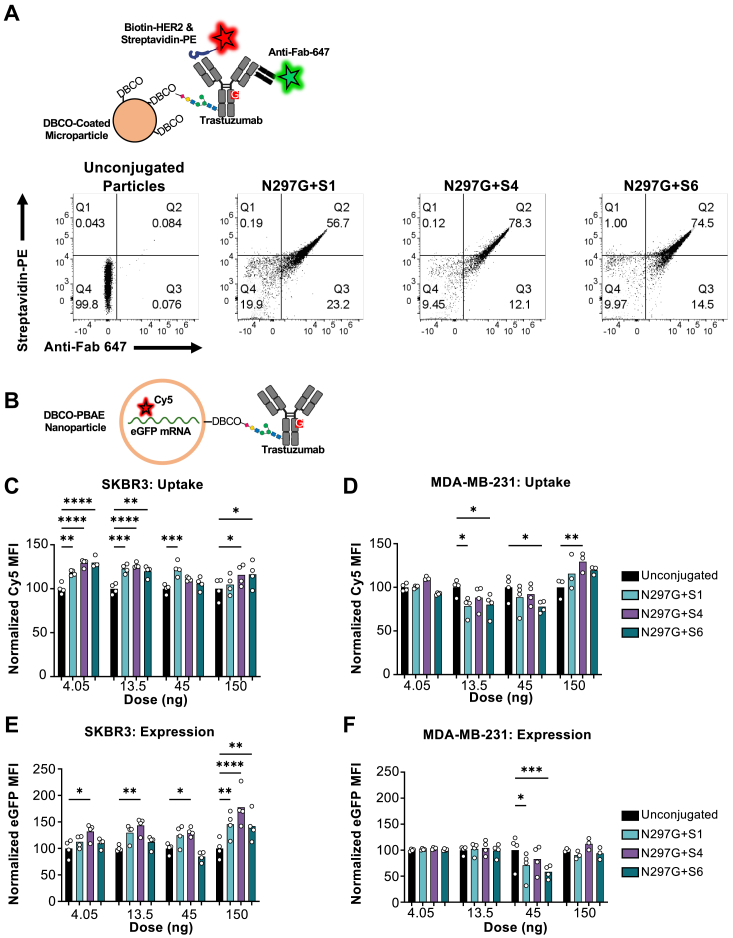
**Fc glycovariant conjugation to biomaterials enables targeted gene delivery.***A*, schematic of detection scheme for flow cytometry analysis of azido-modified trastuzumab glycovariants linked to DBCO-coated magnetic microparticles. Antibody conjugation is detected with a fluorescent anti-Fab antibody and target antigen binding is detected using biotinylated HER2 and secondary fluorescent streptavidin staining. Representative flow cytometry plots are shown below. *B*, cartoon depicting poly(beta-amino ester) (PBAE) nanoparticle encapsulating cyanine-5 (Cy5)-labeled enhanced *green* fluorescent protein (eGFP)-encoding mRNA conjugated to trastuzumab glycovariant antibodies. *C* and *D*, nanoparticle uptake in transfected HER2^+^ SKBR3 (*C*) and HER2^-^ MDA-MB-231 (*D*) cells, normalized to the unconjugated control for each concentration. *E* and *F*, eGFP expression in transfected SKBR3 (*E*) and MDA-MB-231 (*F*) cells, normalized to the unconjugated control for each concentration. Statistical significance was determined by two-way ANOVA with a Dunnett *post hoc* test. ∗*p* ≤ 0.05, ∗∗*p* ≤ 0.01, ∗∗∗*p* ≤ 0.001, and ∗∗∗∗*p* ≤ 0.0001 (n = 4).

To further elucidate the potential for our glycoengineering strategy in therapeutic applications, we sought to conjugate our engineered glycovariants to biomaterials scaffolds, specifically polymer-based particles. Such antibody-biomaterials conjugates have vast potential in targeted therapeutic approaches such as biomimetic modulators and gene delivery platforms. As proof of concept, we first chose to conjugate our glycoengineered antibodies to DBCO-coated magnetic microparticles since the size of these particles allows detection by flow cytometry. We found that each of the azido-modified S1, S4, and S6 trastuzumab glycovariants was successfully loaded onto DBCO-coated microparticles while retaining the ability to bind soluble HER2 (Fig. 6*A*). We next looked to conjugate our glycovariant antibodies to polymeric nanoparticles as a means of targeting the transfection of genetic cargo. We employed poly(beta-amino ester) (PBAE)-based nanoparticles, which have been used extensively for nucleic acid delivery (35). DBCO-modified PBAE nanoparticles encapsulating a cyanine-5 (Cy5)-labeled mRNA encoding enhanced green fluorescent protein (eGFP) were conjugated to azido-modified S1, S4, and S6 trastuzumab glycovariants, and the resulting nanoparticle conjugates were applied to cells for monitoring of uptake (*via* Cy5 fluorescence) and gene delivery (*via* eGFP fluorescence) (Fig. 6*B*). We observed that antibody-conjugated nanoparticles containing each of the three trastuzumab glycovariants showed increased uptake compared to unconjugated control nanoparticles in HER2-expressing SKBR3 cells (Fig. 6*C*), whereas antibody-conjugated particles exhibited equivalent or lower uptake compared to unconjugated control nanoparticles in HER2^-^ MDA-MB-231 cells (Fig. 6*D*). Furthermore, the antibody-conjugated nanoparticles led to higher gene expression relative to unconjugated control nanoparticles, most prominently at the 150 ng dose, in SKBR3 cells (Fig. 6*E*), whereas the antibody-conjugated nanoparticles led to lower or equivalent expression relative to unconjugated control nanoparticles at all concentrations with the exception of the 150 ng dose in MDA-MB-231 cells (Fig. 6*F*). Taken together, these findings establish that our platform is compatible with conjugation to nanoparticles and can be used for targeted gene delivery applications.

### Engineered Fc glycovariant antibodies show promising developability properties

With an eye towards clinical translation of our engineered glycovariant antibodies, we thoroughly evaluated several developability properties of the glycovariants and their respective conjugates. To evaluate the stability of the glycan linkage, we performed stability studies in human plasma using fluorescent dye-conjugated S1, S4, and S6 trastuzumab glycovariants. Over the course of 8 days, we did not observe decay in the fluorescent signal (Fig. S6*A*), indicating that this linkage is stable under physiological conditions. We additionally measured the thermal stability of trastuzumab glycovariants and their respective MMAE-conjugated ADCs. The single mutant S4 and S6 trastuzumab glycovariants had similar melting temperatures to the N297G trastuzumab antibody (Fig. S6*B*, Table S6). The single mutant S1 trastuzumab glycovariant and the triple mutant S146 glycovariant showed slightly lower melting temperatures but still exceeded 54 °C. Importantly, due to the very mild conditions required for conjugation, the thermal stability was not affected by drug conjugation, as no significant differences in melting temperature were observed between each glycovariant antibody and its respective ADC (Fig. S6*B*, Table S6). DLS also indicated that the installed glycans did not induce aggregation in any of the trastuzumab glycovariants that were tested (Fig. S6*C*). Altogether, these biophysical assays demonstrate that our glycoengineering platform leads to the stable attachment of glycans and does not adversely affect antibody developability. Nonetheless, our biophysical studies showed an attenuated level of binding to FcRn and reduced Tm1 for the S146 glycovariant; thus, we wondered whether these undesired effects could be corrected by incorporating substitutions designed to enhance these parameters. To this end, the FcRn affinity-enhancing substitutions M252Y/S254T/T256E (YTE) (36) and the stabilizing engineered disulfide R292C/V302C (SEFL) (29) were simultaneously introduced into the S146 trastuzumab glycovariant (N297G + YTE  + SEFL + S146) to improve FcRn affinity and thermal stability, respectively. A corresponding aglycosylated variant N297G + YTE  + SEFL was also produced. Interestingly, while the introduction of YTE improved the affinity of the aglycosylated variant for human FcRn, these substitutions increased the binding level of the S146 trastuzumab glycovariant to that of the N297G trastuzumab antibody but did not alter its affinity (Fig. S6*D*, Table S3). In addition, the incorporation of the engineered disulfide (SEFL) increased the Tm1 of the triple-site S146 trastuzumab glycovariant by about 5 °C to ∼60 °C (Table S6). The compatibility of these substitutions with engineered glycosylation sites illustrates the modularity of this platform, and that various other IgG engineering strategies can be combined with this approach to further improve the developability of Fc glycovariants.

To further characterize our engineered glycovariants, we probed the glycan linkages formed through DBCO-based conjugation by treatment of dye-conjugated S1, S4, and S6 trastuzumab glycovariant antibodies with various glycosidases. As expected, PNGase digestion (which cleaves all N-linked glycans) eliminated all detectable fluorescence for each of the glycovariant antibodies (Fig. S7*A*). From a translatability standpoint, the ability of circulating sialidases to cut azido-modified sialic acids from glycoconjugates raises the concern that ADCs made using this platform would be unstable *in vivo*. This concern was allayed, however, by our finding that all 3 dye-conjugated trastuzumab glycovariants were resistant to the α2-3,6,8,9 bacterial sialidase when administered post-conjugation (Fig. S7*A*). We suspect that fluorophore linkage may obstruct recognition of the sugar analog by this sialidase. To demonstrate that the incorporated glycans were indeed sialic acids, we pre-treated unconjugated protein with sialidase and then subjected the material to DBCO-fluorophore conjugation. This treatment regimen led to minimal dye conjugation for all three trastuzumab glycovariant antibodies (Fig. S7*B*), demonstrating that the azide groups within the glycovariants were incorporated as sialic acids. Considering the resistance to bacterial sialidase post-conjugation, we investigated whether these linkages were also resistant to the human sialidase neuraminidase 1, which would be advantageous for the plasma stability of the glycan linkage. Encouragingly, we observed that all 3 dye-conjugated trastuzumab glycovariants were resistant to cleavage by this sialidase as well (Fig. S7*C*). Overall, we verified that our newly installed conjugation was indeed site-specific and that the glycan linkages were robust to human sialidase cleavage, potentially boosting plasma stability.

## Discussion

There is a high demand for antibody conjugates in research, industrial, and medical applications, yet state-of-the-art techniques for molecular conjugation to antibodies suffer from drawbacks that affect the ability to generate stable and developable molecules in a rapid and reproducible manner. These drawbacks include impaired antibody yield, structure, and stability due to exposure to harsh chemical reactions, complicated manufacturing of the required chemical reagents and/or enzymes, and the need for technically demanding processing and purification steps by the user (6, 7). Glycoengineering platforms can potentially overcome these limitations by combining protein, genetic, and metabolic engineering to manipulate the functionality of natural proteins to suit various use cases (10). Notably, metabolic glycoengineering presents a largely untapped manufacturing technique through which modified, non-natural carbohydrates introduce chemically selective groups for conjugation, including soluble, recombinant proteins.

In this study, we combined metabolic engineering and protein engineering strategies to develop a simple but powerful conjugation platform. Specifically, we introduced novel N-linked glycosylation sites into the Fc region of hIgG1, which is the most commonly deployed scaffold for therapeutic antibodies. We developed several glycovariant antibodies that were produced robustly with high purity from mammalian cell expression systems, while also recapitulating the binding and functional activities of their respective parent molecules. Purification of these Fc glycovariant antibodies in the presence of azido-functionalized sugar analogs led to solvent-accessible azide groups that could be conjugated to DBCO-containing molecules for a variety of applications using well-established click chemistry procedures. Moreover, our system showed impressive versatility, as it was effective for three antibodies of distinct specificities. As an initial example of the utility of our platform, we demonstrated that this approach could be used for direct fluorescent labeling of antibodies; this concept could be easily extended to conjugate antibodies to other detection agents, for instance, quantum dots or radionuclides (4, 5), for both research and translational applications. We showed that our system was compatible with multiple standard mammalian expression cell lines (HEK 293F, Expi293F, CHO-S, and ExpiCHO), with the ability to tune the extent of glycan incorporation in these systems. Furthermore, the supplementation of both ManNAz and GalNAz analogs could be used to modulate the total number of azides available for conjugation. Moreover, we demonstrated that up to three distinct sites in the Fc domain can be mutated simultaneously to introduce novel N-glycans, offering an additional opportunity to toggle the extent of conjugation to suit the desired application. Importantly, glycan-mediated conjugation is stable in human plasma, and our approach yields constructs with similar thermal stability and quality to unconjugated parental antibodies, offering promise for their commercial or medical developability.

As ADCs and antibody-biomaterial conjugates are growing therapeutic modalities, there is increasing demand for straightforward, inexpensive, and efficient approaches to antibody conjugation across the drug development landscape. Current clinical ADCs make use of either amine chemistry, which can lead to non-specific and heterogeneous labeling of lysine residues throughout a protein, or thiol chemistry, which requires partial reduction of the antibody that may disrupt intrinsic disulfides that enforce chain assembly (6, 7, 31, 34). Our Fc glycoengineering platform circumvents these challenges through site-specific modification without requiring harsh chemical reagents that disturb antibody structure. We demonstrated the application of our platform to generate conjugates incorporating the clinically approved anti-HER2 antibody, trastuzumab, which is used in several clinical ADCs (1, 30, 31). Our single mutant trastuzumab glycovariants conjugated with the anti-mitotic agent MMAE carried sufficient drug payload to achieve selective and efficient killing of HER2-expressing breast cancer cells. Moreover, by simultaneously incorporating 3 novel N-linked glycosylation sites into the Fc domain, we increased the payload sufficiently to achieve remarkably potent (sub-nanomolar IC_50_) cytotoxicity on multiple HER2^+^ cell lines. We additionally showed that our glycoengineering-based conjugation approach enables functional attachment of antibodies to microparticles as well as polymeric nanoparticles used as gene delivery vehicles. Collectively, these findings indicate that our platform could be integrated with the many biomedical and biotechnological systems that now use biorthogonal click chemistries (37).

Overall, we successfully demonstrated that combining high-flux ManNAc analogs with engineered N-linked glycans in the Fc region can serve as a modular and effective platform for molecular conjugation to antibodies. The simplicity and generalizability of this approach make it a promising strategy for producing antibody conjugates with reduced concern for deleterious effects on their function and developability compared to extant non-glycan-based conjugation strategies. Subsequent research could scan the entire Fc domain for further conjugation sites, which could be installed independently or in combination with the sites we report herein to tune the extent of conjugation. We also envision that certain extensions of our workflow will not require the discovery or introduction of new N-linked glycosylation sites, for instance in cases wherein these sites can be grafted onto backbones of similar sequence (*e.g.*, mouse IgG) or for other proteins beyond IgGs in which grafting is not needed since natural glycosylation is present. A vast array of enzymes, cytokines, growth factors, and other proteins with inherent glycosylation sites may only require supplementation with sugar analogs during the protein production pipeline for the installation of azido groups.

In addition to being superior to current conjugation strategies that use non-glycan moieties found in antibodies, such as amine or thiol groups, our strategy has advantages over alternative glycoengineering methods used for antibody conjugation. For example, several alternative glycoengineering conjugation workflows require specialized enzymes and custom-made synthetic linkers, employing a transglycosidase that concurrently cleaves the canonical N297 Fc glycan and replaces it with an azido-functionalized linker (38, 39, 40, 41, 42, 43, 44). By contrast to this strategy, our platform leverages the natural cell glycosylation machinery to install chemical functional groups and does not require post-purification manipulation for installing conjugation sites. A key advance in the current work over previous efforts to use metabolic engineering to conjugate antibodies is our use of our newly introduced glycan sites to increase valency. For example, efforts to target the single fucose present in the N297 Fc glycan (17) are limited by a maximum theoretical drug-to-antibody ratio (DAR) of 2 since each Fc glycan in an IgG dimer has a single fucose. Targeting sialic acid can provide a higher DAR (up to 4) because Fc glycans are biantennary and each terminus can be capped with this monosaccharide; in practice; however, Fc glycans are poorly sialylated (typically <1–2%) making this strategy non-viable. One strategy to circumvent the poor sialylation of canonical Fc glycans is to target O-glycans that are not buried between the two protein chains and are therefore more accessible for sialylation or can be targeted with azido-modified GalNAc (45). O-glycans, however, rarely occur in antibodies and it is difficult to install this form of glycosylation because of a lack of a reliable consensus sequon (46). Accordingly, we focused on N-glycans and identified multiple glycosylation sites that could be reproducibly added to the Fc region of IgG antibodies alone or in combination. We consider the work we report here, which already reproduces DARs of existing ADCs of ∼2 to 3, to be a foundation for higher valency ADCs in the future. In particular, the six added N-glycans in an IgG dimer have the potential to display from 12 (if the glycans are bivalent) to 24 (if the glycans are tetravalent) azido-functionalized sialic acids. A current limitation, however, is that only a small fraction, reportedly 2 to 20%, of natural sialic acids are replaced with their non-natural counterparts (47), which is consistent with our current DAR of ∼2. In the future, the use of UDP-N-acetylglucosamine 2-epimerase (GNE) knockout cells that do not have a flux of endogenously produced ManNAc into the sialic acid biosynthetic pathway and therefore can incorporate non-natural analogs with 70% or more efficiency (48) offers the potential for much higher DARs.

We also anticipate that the tunability of this platform will offer significant benefits, as we have shown that the payload can be tailored to the application through modification of the cellular expression system, substitution or combination of different sugar analogs, or changing the number of N-linked glycosylation sites inserted. Future efforts will investigate how overexpression or inhibition of various glycoenzymes (*e.g.*, GNE, which can increase non-natural analog incorporation (48), or over-expressed sialyltransferases, which can increase sialic acid incorporation into recombinant antibodies (49, 50)), can be used to enhance azide incorporation. Additionally, future work will explore how different click chemistries can be incorporated for distinct use-cases. Looking ahead, our platform can inspire new glycoengineering approaches that will further expand the protein conjugation toolkit.

## Experimental procedures

### Novel N-linked site design

Amino acid residues distributed throughout the heavy chain constant domains 2 and 3 (CH2 and CH3) of the human immunoglobulin G1 (hIgG1) fragment crystallizable (Fc) region were chosen for the installation of novel sites of N-linked glycosylation. N-linked glycan insertion sites can be created by the introduction of an N-X-S/T consensus sequence into a protein, where X is any amino acid except proline (8). A sliding window method was used to identify all potential sites for insertion of this consensus sequence. Theoretical mutations were chosen to either install N-X-T, or N-X-S, whichever required fewer substitutions. Structures of candidate glycovariants were then modelled using PyRosetta (19), based on the wild-type hIgG1 Fc structure (PDB ID: 5JII) (20). Final sites were chosen according to the following criteria: high solvent exposure, flexible loop secondary structure (determined by DSSP algorithm), minimal disruption to the native sequence, and predicted probability of N-glycosylation >0.6 as determined by the NetNGlyc server (18).

### Analog design and antibody production

Theoretically, various chemical groups can be functionally introduced for conjugation. We elected to provide azido-modified carbohydrate analogs due to the commercial availability of dibenzocyclooctyne (DBCO) moiety-activated molecules, thereby avoiding the need for copper-catalyzed “click chemistry” conjugation reactions. The synthesis of 1,3,4-O-Bu_3_ManNAz and has been previously described (11, 51). Following synthesis and purification, a concentrated solution (100 mM) of each analog was made using 200 proof ethyl alcohol for long term storage −80 °C. On the day of antibody transfection, and every 2 days thereafter, 1,3,4-O-Bu_3_ManNAz solution was added to cell cultures at a final concentration of 100 μM unless noted otherwise.

The heavy chain (HC) sequences and light chain (LC) sequences for the hIgG1 lambda F5111 antibody were separately cloned into the gWiz vector (Genlantis) (21). Similarly, chimeric HC and LC sequences for an anti-mouse CD19 antibody containing the variable domains of the rat IgG2a 1d3 (52) antibody and the constant hIgG1 HC and kappa LC domains were separately cloned into the gWiz vector. The HC and LC sequences for the hIgG1 kappa trastuzumab antibody were separately cloned into the gWiz vector https://www.genome.jp/dbget-bin/www_bget?D03257+D09980 (Accessed July 7, 2024). Site-directed mutagenesis to create plasmid vectors containing mutated DNA sequences encoding glycovariant HCs was performed as previously described (53). The FcRn affinity-enhancing substitutions M252Y/S254T/T256E (YTE) (36) and stabilizing engineered disulfide R292C/V302C (SEFL) (29) and were simultaneously introduced into the S146 trastuzumab glycovariant to improve FcRn affinity and thermal stability, respectively. For antibody production, the HC and LC DNA plasmids constructs were co-transfected at a 1:1 mass ratio into human embryonic kidney (HEK) 293F cells at a density of 1 × 10^6^ cells/ml or Expi293F cells at a density of 2 × 10^6^ cells/ml using polyethylenimine (PEI) MAX (Polysciences). In brief, DNA (1 mg/L culture) and PEI MAX (5.3 mg/L culture) were diluted in Opti-MEM (100 ml/L of culture) (Thermo Fisher), briefly vortexed, and incubated at room temperature for 10 min. Subsequently, the DNA/PEI MAX mixture was added to the culture with gentle swirling. Transfection of Chinese hamster ovary (CHO)-S cells was performed using the poly(beta-amino ester) (PBAE) formulation 4-4-6, as previously described (54). The day after the transfection of Expi293F cells, valproic acid, sodium propionate, and glucose (Sigma) were added to final concentrations of 5 mM, 6.9 mM, and 46 mM, respectively. Secreted antibodies were purified from cell supernatants 4 to 6 days post-transfection *via* protein G agarose affinity chromatography. A stable (CHO-S-derived) ExpiCHO cell pool expressing the S146 trastuzumab glycovariant (with the N297G substitution) and the sialyltransferase ST6GAL1 was generated using a custom plasmid containing three expression cassettes (heavy chain, light chain, and ST6GAL1-IRES-PuroR) and PiggyBac transposition, as previously (55, 56). Production using the stable pool was performed by seeding cells in fresh ExpiCHO Stable production media at 3 × 10^6^ cells/ml, immediately shifting the temperature to 31 °C, supplementing with 200 μM 1,3,4-O-Bu3ManNAz and 4 g/L glucose every 2 days, and harvesting 7 days after seeding. Purification proceeded as above *via* protein G agarose affinity chromatography.

### Cell culture

HEK293F, Expi293F, CHO-S, and ExpiCHO cells (Thermo Fisher) were supplemented with 2 U/ml penicillin, 2 μg/ml streptomycin (Gibco) and cultured in serum-free media according to the manufacturer’s instructions. A20 B cells were cultured in ATCC-Modified RPMI-1640 (Thermo Fisher) supplemented with 10% fetal bovine serum (FBS), 100 U/ml penicillin, 100 μg/ml streptomycin (Gibco), and 0.05 mM 2-mercaptoehanol. SKBR3 cells were cultured in McCoy’s 5a Medium (Thermo Fisher) supplemented with 10% FBS, and 100 U/ml penicillin, 100 μg/ml streptomycin (Gibco). MDA-MB-453 and MDA-MB-231 cells were cultured in Leibovit’s L-15 Medium (Thermo Fisher) supplemented with 10% FBS, and 100 U/ml penicillin, 100 μg/ml streptomycin (Gibco). HCC1954 cells were cultured in ATCC-Modified RPMI-1640 (Thermo Fisher) supplemented with 10% FBS and 100 U/ml penicillin-streptomycin (Gibco). All cell lines were maintained at 37 °C in a humidified atmosphere with 5% CO_2_.

### Fluorescent dye and drug conjugation

Purified antibodies were conjugated overnight at 4 °C in phosphate-buffered saline (PBS, pH 7.4) containing 20 M excess of BP Fluor 647 DBCO (BroadPharm). Excess dye was removed through repeated buffer exchange in a 30 kDa MWCO centrifugal filter (Amicon). The Dye/Protein ratio was then quantified using UV/Vis spectroscopy with a NanoDrop (ε_648nm,dye_ = 270,000 M^−1^cm^−1^). For conjugation of MMAE, DBCO-PEG4-Val-Cit-MMAE (BroadPharm) was similarly incubated with antibodies in 20 M excess overnight and removed through centrifugal filtration as described above.

### Binding studies

For biolayer interferometry studies using an Octet instrument, biotinylated human interleukin-2 (IL-2), produced *via* HEK 293F cell secretion, as previously described (15), human neonatal Fc receptor (FcRn) (Sino Biological), human Fcγ receptor I (FcγRI) (Sino Biological), human FcγRIIa (Sino Biological), and human epidermal growth factor receptor 2 (HER2) (Sino Biological) were immobilized to streptavidin-coated biosensors (Sartorius). For IL-2 and HER-2 binding studies, a background buffer of phosphate-buffered saline pH 7.4 containing 0.1% (w/v) bovine serum albumin (PBSA) was used, and tips were regenerated in 0.1 M glycine pH 2.7. For FcRn binding studies, a background buffer of PBSA pH 5.6 was used, and tips were regenerated in PBSA pH 7.4. For FcγR binding studies, a background buffer of PBSA pH 7.4 was used, and tips were regenerated in 0.1 M glycine pH 3.5.

For mouse CD19^+^ (mCD19^+^) cell staining studies, A20 cells were incubated with 1 μg/ml of fluorescently labeled antibody at 4 °C for 20 min. Cells were then washed twice with PBSA and analyzed on a Cytoflex flow cytometer (Beckman Coulter).

### Cytotoxicity assays

The day before treatment, cells were seeded at 5000/well in 100 μl complete media in a tissue culture-treated 96-well flat bottom plate (Corning). On the day of the experiment, 10 μl of antibody conjugated with monomethyl auristatin E (MMAE) or DBCO-PEG4-Val-Cit-MMAE alone was added in quadruplicate. 96 h after treatment, cell viability was determined by XTT assay (Thermo Fisher) according to the manufacturer’s protocol.

### Microparticle binding studies

For conjugation of DBCO magnetic microparticles (VectorLaboratories), 150 μg of particles were incubated with 1 μg of Ab overnight at 4 °C. Particles were then washed with PBSA and stained with 50 nM biotinylated HER2 (Sino Biological) for 20 min at room temperature. Particles were then washed with PBSA and stained with a 1:50 dilution of phycoerythrin (PE)-conjugated streptavidin (Thermo Fisher) and a 1:200 dilution of 647-conjugated Alexa Fluor 647 AffiniPure Fab Fragment Goat Anti-Human IgG (H  + L) (JacksonImmunoResearch) in PBSA for 20 min on ice. The beads were then washed twice with PBSA and analyzed on a Cytoflex flow cytometer.

### Nanoparticle preparation and transfection

A PBAE was synthesized as described previously (57). Briefly, bisphenol A glycerolate (1 glycerol/phenol) diacrylate (Sigma-Aldrich) was mixed with 1-dodecylamine (from Alfa Aesar) and 4-(2-aminoethyl)morpholine (Sigma-Aldrich) (80:20 mol/mol ratio of amines, 2.3:1 mol/mol ratio of vinyl groups to amine) in a 600 mg/ml solution in anhydrous dimethylformamide. The reaction was allowed to proceed with stirring at 85 °C for 48 h. The polymer was diluted to 200 mg/ml in anhydrous tetrahydrofuran and end-capped with diethylentriamine (EMD Millipore) at room temperature for with stirring 2 h. The end-capped PBAE was precipitated and washed in diethyl ether, then dried under vacuum at room temperature for 48 h. The purified PBAE was dissolved in anhydrous dimethyl sulfoxide and stored at −20 °C.

To measure both uptake and gene expression simultaneously, enhanced green fluorescent protein (eGFP) mRNA (CleanCap, 5-methoxyuridine-modified, Trilink Biotchnologies) was labeled with Cy5 using the Label IT Nucleic Acid Labeling Kit (Mirus Bio) according to the manufacturer's instructions. The Cy5-labeled eGFP mRNA was mixed with unlabeled eGFP mRNA at a 1:4 ratio for nanoparticle (NP) formulation.

Antibody was conjugated to 1,2-dimyristoyl-rac-glycero-3-methoxypolyethylene glycol-2000-dibenzocyclooctyne (DMG-PEG2k-DBCO) (Nanocs) by incubation overnight at 4 °C with an azide-functionalized antibody. mRNA-loaded PBAE NPs were formed by self-assembly. mRNA was diluted in sodium acetate buffer (NaAc, pH 5), and the PBAE was dissolved in a 90:10 (vol/vol) mixture of ethanol and NaAc. The polymer solution was mixed 1:1 (vol/vol) at a 30:1 mass ratio of polymer to mRNA to form nanoparticles, which were dialyzed against 0.25 mM NaAc using a dialysis cassette with a 50 kDa molecular weight cutoff for 1 h. The salt concentration and pH were raised by the addition of 10 × PBS to a final concentration of 0.5 × PBS, and the NPs were allowed to incubate for 30 min before being stabilized by addition of DMG-PEG2k or DMG-PEG-antibody to a final mass ratio of 1:10 DMG-PEG:PBAE. Low-endotoxin sucrose was added to the NPs as a cryoprotectant at a final concentration of 50 mg/ml, and the particles were stored at −80 °C until use. The final NPs contained mRNA at a concentration of 0.02 mg/ml.

Cells were seeded in complete medium 1 day before transfection to allow adhesion (10,000 cells/well in 96-well plates). On the day of transfection, NPs were thawed, diluted in complete medium, and added to cells at a final dosage of 4.05 ng, 13.5 ng, 45 ng, or 150 ng/well in 120 μl volume. Cells were incubated for 24 h with the NPs, then trypsinized and analyzed by flow cytometry (Attune N x T, Thermo Fisher) for Cy5 fluorescence and eGFP expression, using 7-aminoactinomycin D (7-AAD) (Thermo Fisher) to exclude dead cells. Experiments were performed in quadruplicate. Statistical significance was determined by two-way ANOVA with a Dunnett *post hoc* test.

### Plasma stability studies

Plasma stability studies of antibody-fluorophore conjugates were performed as previously described with minor modifications (58). For each time point, 500 ng of each BP Fluor 647-labeled antibody was incubated in 66% plasma at 37 °C. At each time point, the sample was diluted with SDS sample buffer and snap frozen. The samples were then subjected to SDS-PAGE analysis and in-gel fluorescent imaging. Percent initial signal was calculated by dividing the fluorescent intensity of the conjugate at each time point by the intensity at the starting point. Experiments were performed in duplicate.

### Thermal stability and purity measurements

Antibody melt curves were obtained using a Protein Thermal Shift Kit (Thermo Fisher), following the manufacturer’s protocol. Antibody melting temperatures were determined by maxima of the differential melt-curve. For analytical high-performance liquid chromatography (HPLC) studies, 10 μg protein was separated on an Acclaim SEC-1000 (7.8 × 300 mm). Dynamic light scattering measurements were collected using a Zetasizer Pro (Malvern).

### Glycosidase treatment

For each enzyme, 500 ng of BP Fluor 647 dye-conjugated antibody was digested. PNGase F and α2,3,6,8,9 Neuraminidase (NEB) treatments were performed according to the manufacturer’s instructions. Human neuraminidase1 (Prospec) cleavage was performed at a 1:1 mass ratio with antibody in 0.1 M sodium acetate, pH 4.5, as previously described (59). Bands were visualized using in-gel fluorescent imaging on an iBright FL1500 (Thermo Fisher) under the AlexaFluor 647 channel.

## Data availability

Data are available from the authors upon request.

## Supporting information

This article contains supporting information.

## Conflict of interest

The authors declare the following financial interests/personal relationships which may be considered as potential competing interests.

Z. J. B., K. D. -B., K. J. Y., and J. B. S. are listed as co-inventors on a patent describing the technologies presented herein.
