# Supplementary material for: Production of site-specific antibody conjugates using metabolic glycoengineering and novel Fc glycovariants[image]
Source: J Biol Chem. 2024 Nov 16;300(12):108005. doi: 10.1016/j.jbc.2024.108005 (PMC11697773; doi:10.1016/j.jbc.2024.108005)
Supplement: Supporting information [file mmc1.pdf]

Supplementary Figure 1

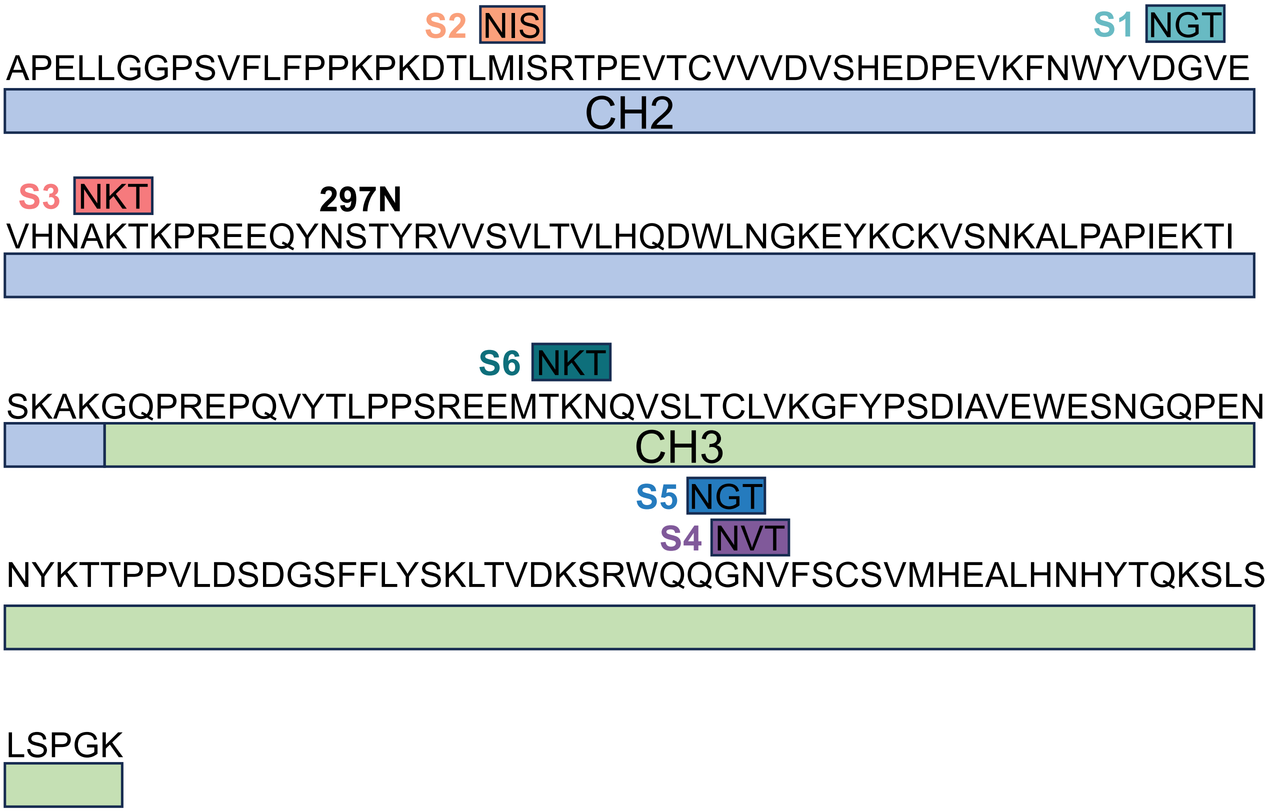

Figure S1: Sequence layout of engineered Fc glycovariants. Annotated amino acid sequence of heavy chain constant 2 (CH2) and CH3 regions of human immunoglobulin G1 (IgG1) fragment crystallizable (Fc) domain, with canonical N297 glycan and engineered glycovariant sites indicated.

Supplementary Figure 2

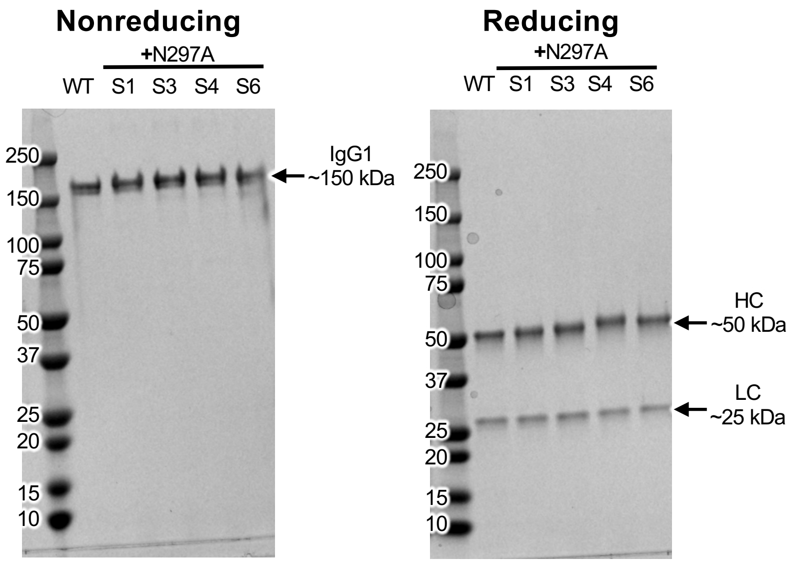

Figure S2: Production of 1d3 antibody and derivative Fc glycovariants. Nonreducing and reducing SDS-PAGE analysis of the 1d3 antibody in wild type (WT) and engineered Fc glycovariant formats. HC, heavy chain; LC, light chain.

Supplementary Figure 3

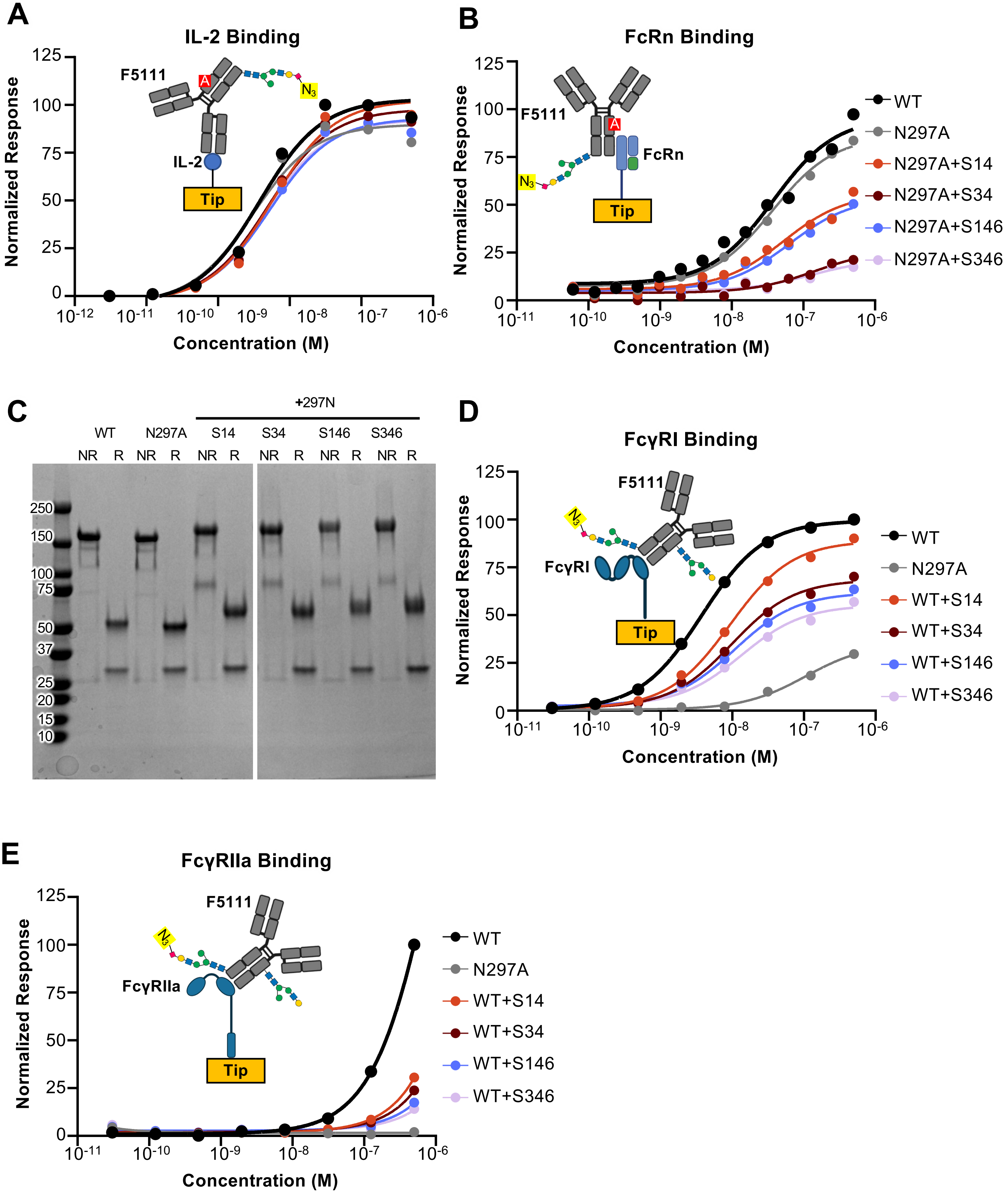

Figure S3: Fc glycovariants with multiple glycosylation sites retain antigen binding and may retain Fc receptor binding. (A-B) Biolayer interferometry studies of the equilibrium binding between immobilized IL-2 (A) or FcRn (B) and soluble double and triple mutant F5111 glycovariants with N297A substitutions. (C) SDS-PAGE analysis of the F5111 antibody in wild type (WT), N297A, and engineered Fc glycovariant formats. (D-E) Biolayer interferometry studies of the equilibrium binding between immobilized FcγRI (D) or FcγRIIa (E) and soluble double and triple mutant F5111 glycovariants with the N297 glycosylation site intact. HC, heavy chain; LC, light chain; NR, Non-reducing; R, reducing.

Supplementary Figure 4

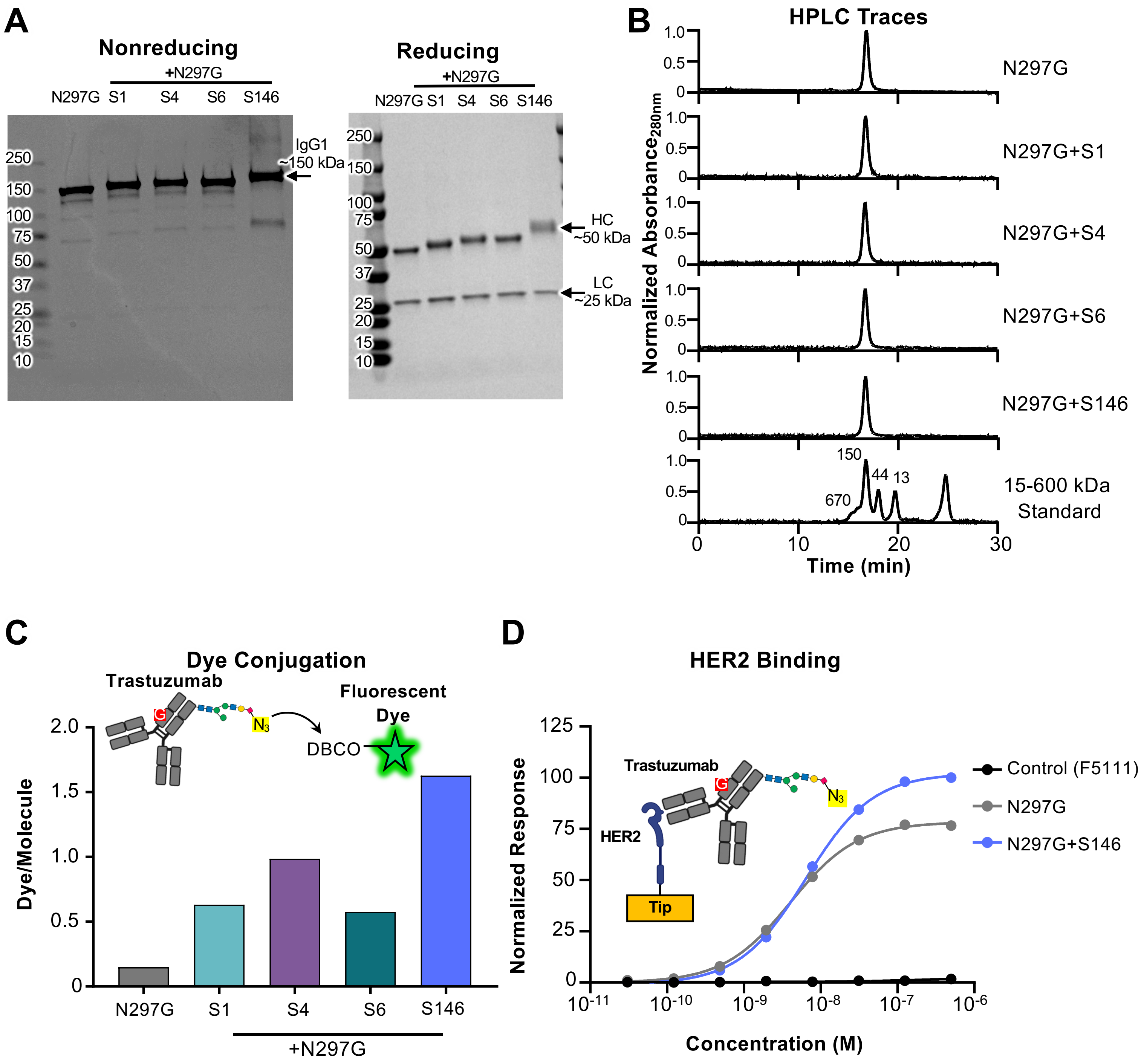

Figure S4: Trastuzumab glycovariants are produced with high purity and incorporate azides to allow fluorescent labeling. (A) Nonreducing and reducing SDS-PAGE analysis of the trastuzumab antibody in N297G, and engineered Fc glycovariant formats. (B) Analytical high-performance liquid chromatography (HPLC) traces of the trastuzumab antibody glycovariants. (C) Average number of dye molecules per antibody molecule, determined by dye/protein ratio of azide-functionalized trastuzumab glycovariant antibodies labeled with dibenzocyclooctyne (DBCO)-linked fluorescent dyes, as measured by UV/Vis spectroscopy. (D) Biolayer interferometry studies of the equilibrium binding between immobilized HER2 and soluble trastuzumab glycovariants.

Supplementary Figure 5

A

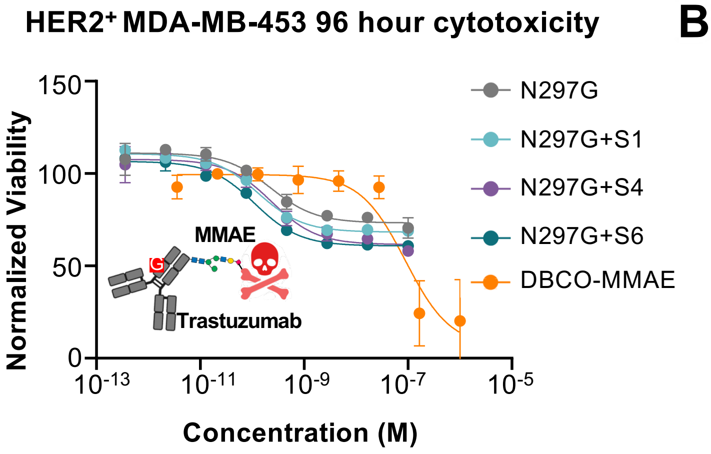

B

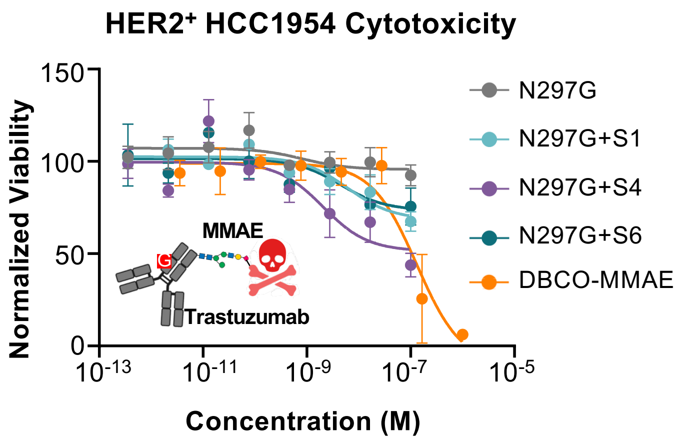

C

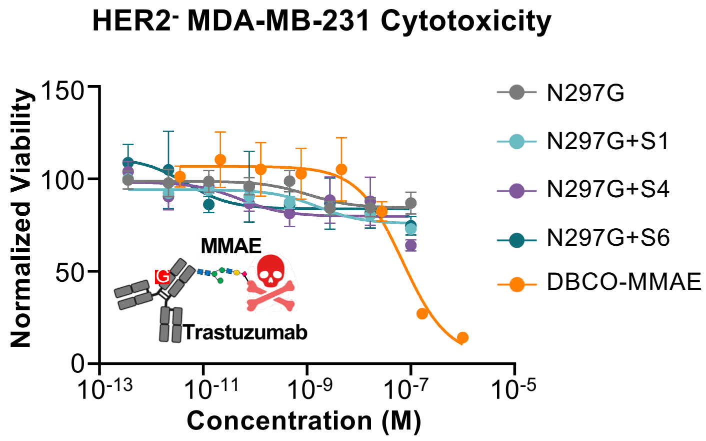

Figure S5: Fc glycovariants demonstrate potent and specific tumor cell killing upon formulation as antibody-drug conjugates. (A-C) Cytotoxicity of single site trastuzumab glycovariant ADCs against $HER2^+$ MDA-MB-453 (A), $HER2^+$ HCC1954 (B), or $HER2^-$ MDA-MB-231 (C) human breast cancer cells. DBCO-linked monomethyl auristatin E (MMAE) was used as the drug payload only control. Error bars represent standard deviation (n=4).

Supplementary Figure 6

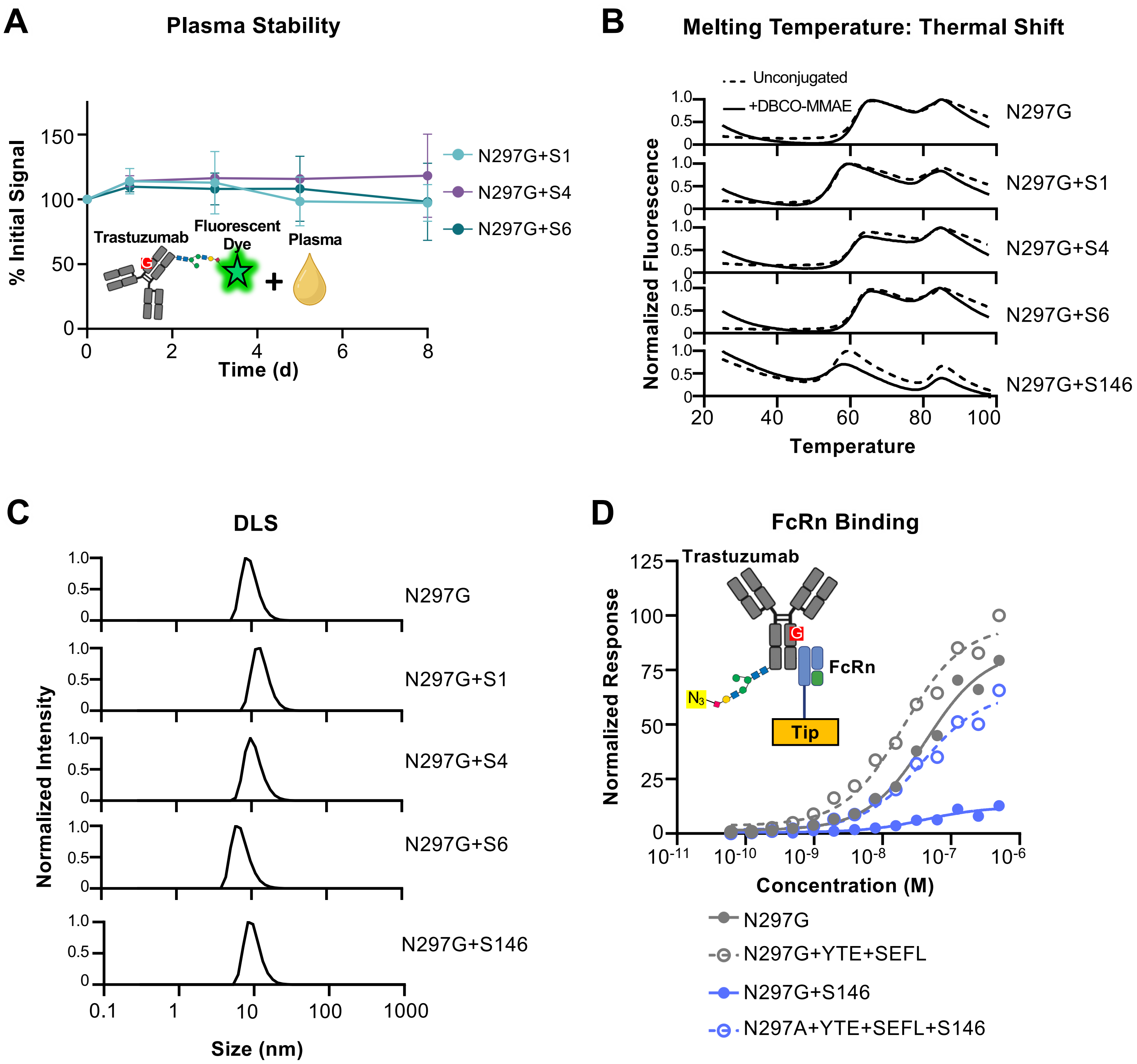

Figure S6: Fc glycovariants show promising developability properties. (A) Plasma stability studies of azide-functionalized trastuzumab glycovariant antibodies labeled with DBCO-linked fluorescent dye. Error bars represent standard deviation (n=2). (B) Thermal shift assays for determination of melting temperatures of trastuzumab glycovariant antibodies in unconjugated and MMAE-conjugated ADC formats. (C) Dynamic-light-scattering (DLS) analysis of trastuzumab glycovariant antibodies. (D) Biolayer interferometry studies of the equilibrium binding between immobilized FcRn and soluble trastuzumab glycovariants with and without the FcRn affinity-enhancing YTE substitution and the stabilizing SEFL substitutions.

Supplementary Figure 7

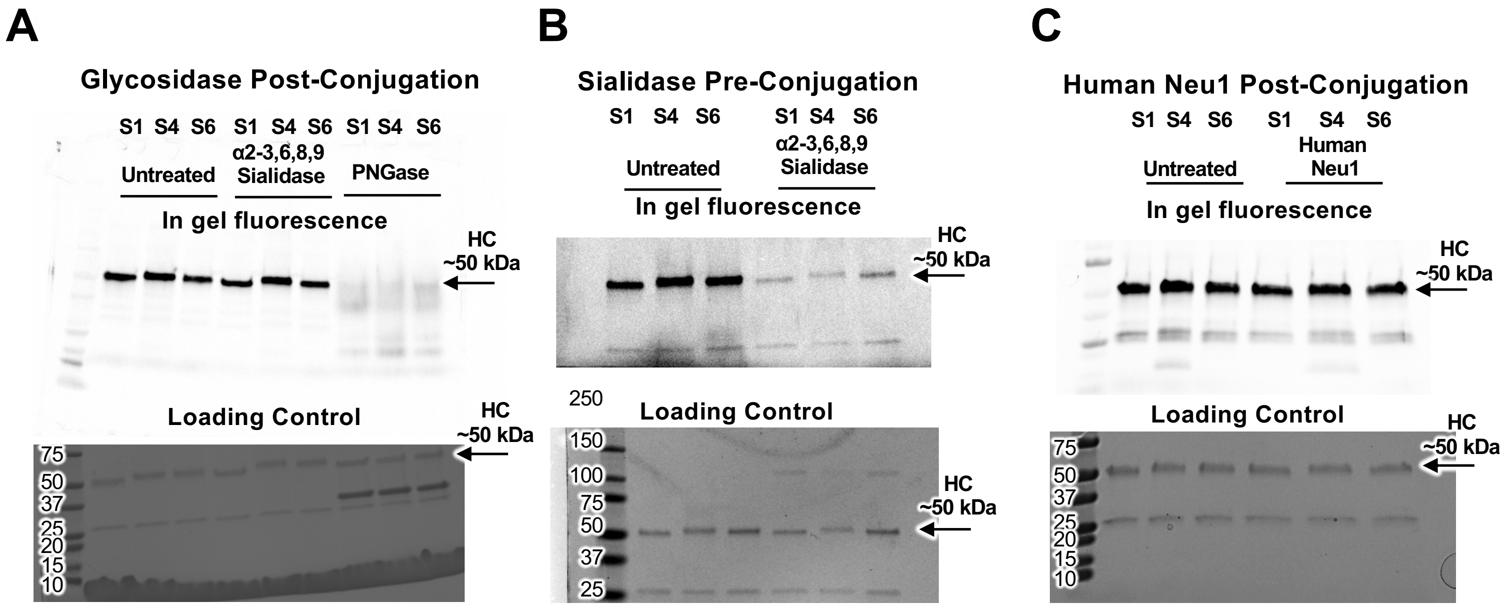

Figure S7: Fc glycoconjugates are formed via sialic acid linkages and are resistant to human sialidase. (A) In-gel fluorescence analysis of DBCO-linked fluorescent dye-labeled trastuzumab glycovariant antibodies following treatment with bacterial sialidase and PNGase. (B) In-gel fluorescence of azido-modified trastuzumab glycovariant antibodies pre-treated with bacterial sialidase then subsequently conjugated to DBCO-linked fluorescent dye. (C) In-gel fluorescence of DBCO-linked fluorescent dye-labeled trastuzumab glycovariant antibodies following treatment with human neuraminidase 1.

Supplementary Tables

Table S1: Fc domain (CH2-CH3) sequences of hIgG1 glycovariants. The installed N-glycan sequons are highlighted in yellow, and the canonical N297 sequon is highlighted in green.

Construct	Sequence
hIgG1 WT	APELLGGPSVFLFPPKPKDTLMISRTPEVTCVVVDVSHEDPEVKFNWYVDGVE<br>VHNAKTKPREEQYNSTYRVVSVLTVLHQDWLNGKEYKCKVSNKALPAPIEKT<br>ISKAKGQPREPQVYTLPPSREEMTKNQVSLTCLVKGFYPSDIAVEWESNGQPE<br>NNYKTTPPVLDSDGSFFLYSKLTVDKSRWQQGNVFSCSVMHEALHNHYTQKS<br>LSLSPGK
hIgG1 N297X	APELLGGPSVFLFPPKPKDTLMISRTPEVTCVVVDVSHEDPEVKFNWYVDGVE<br>VHNAKTKPREEQY(A/G/D/Q)STYRVVSVLTVLHQDWLNGKEYKCKVSNKAL<br>PAPIEKTISKAKGQPREPQVYTLPPSREEMTKNQVSLTCLVKGFYPSDIAVEWE<br>SNGQPENNYKTTPPVLDSDGSFFLYSKLTVDKSRWQQGNVFSCSVMHEALHN<br>HYTQKSLSLSPGK
hIgG1 S1	APELLGGPSVFLFPPKPKDTLMISRTPEVTCVVVDVSHEDPEVKFNWYVNGTE<br>VHNAKTKPREEQY(N/A/G/D/Q)STYRVVSVLTVLHQDWLNGKEYKCKVSNKA<br>LPAPIEKTISKAKGQPREPQVYTLPPSREEMTKNQVSLTCLVKGFYPSDIAVEW<br>ESNGQPENNYKTTPPVLDSDGSFFLYSKLTVDKSRWQQGNVFSCSVMHEALH<br>NHYTQKSLSLSPGK
hIgG1 S2	APELLGGPSVFLFPPKPKDTLNISRTPEVTCVVVDVSHEDPEVKFNWYVDGVE<br>VHNAKTKPREEQY(N/A/G/D/Q)STYRVVSVLTVLHQDWLNGKEYKCKVSNKA<br>LPAPIEKTISKAKGQPREPQVYTLPPSREEMTKNQVSLTCLVKGFYPSDIAVEW<br>ESNGQPENNYKTTPPVLDSDGSFFLYSKLTVDKSRWQQGNVFSCSVMHEALH<br>NHYTQKSLSLSPGK
hIgG1 S3	APELLGGPSVFLFPPKPKDTLMISRTPEVTCVVVDVSHEDPEVKFNWYVDGVE<br>VHNNKTKPREEQY(N/A/G/D/Q)STYRVVSVLTVLHQDWLNGKEYKCKVSNKA<br>LPAPIEKTISKAKGQPREPQVYTLPPSREEMTKNQVSLTCLVKGFYPSDIAVEW<br>ESNGQPENNYKTTPPVLDSDGSFFLYSKLTVDKSRWQQGNVFSCSVMHEALH<br>NHYTQKSLSLSPGK
hIgG1 S4	APELLGGPSVFLFPPKPKDTLMISRTPEVTCVVVDVSHEDPEVKFNWYVDGVE<br>VHNAKTKPREEQY(N/A/G/D/Q)STYRVVSVLTVLHQDWLNGKEYKCKVSNKA<br>LPAPIEKTISKAKGQPREPQVYTLPPSREEMTKNQVSLTCLVKGFYPSDIAVEW<br>ESNGQPENNYKTTPPVLDSDGSFFLYSKLTVDKSRWQQGNVTSCSVMHEALH<br>NHYTQKSLSLSPGK
hIgG1 S5	APELLGGPSVFLFPPKPKDTLMISRTPEVTCVVVDVSHEDPEVKFNWYVDGVE<br>VHNAKTKPREEQY(N/A/G/D/Q)STYRVVSVLTVLHQDWLNGKEYKCKVSNKA<br>LPAPIEKTISKAKGQPREPQVYTLPPSREEMTKNQVSLTCLVKGFYPSDIAVEW<br>ESNGQPENNYKTTPPVLDSDGSFFLYSKLTVDKSRWQNGTVFSCSVMHEALH<br>NHYTQKSLSLSPGK
hIgG1 S6	APELLGGPSVFLFPPKPKDTLMISRTPEVTCVVVDVSHEDPEVKFNWYVDGVE<br>VHNAKTKPREEQY(N/A/G/D/Q)STYRVVSVLTVLHQDWLNGKEYKCKVSNKA<br>LPAPIEKTISKAKGQPREPQVYTLPPSREEMNKTQVSLTCLVKGFYPSDIAVEW<br>ESNGQPENNYKTTPPVLDSDGSFFLYSKLTVDKSRWQQGNVFSCSVMHEALH<br>NHYTQKSLSLSPGK

Table S2: Equilibrium dissociation constant values (K_D) for antibody/antigen binding.

**Antibody Construct**	**K_D (nM) (95% CI) for Target Antigen Binding**
F5111 WT	5.1 (3.4,7.5)
F5111 N297A	4.1 (2.7,6.4)
F5111 N297A+S1	4.2 (2.8,6.6)
F5111 N297A+S2	5.3 (3.7,7.5)
F5111 N297A+S3	4.3 (2.9,6.6)
F5111 N297A+S4	4.2 (2.7,6.4)
F5111 N297A+S5	3.4 (2.1,5.5)
F5111 N297A+S6	4.1 (2.7,6.3)
F5111 N297A+S14	5.7 (3.4,9.7)
F5111 N297A+S34	5.1 (3.1,8.4)
F5111 N297A+S146	5.3 (3.1,9.1)
F5111 N297A+S346	4.7 (2.9,7.7)
Trastuzumab N297G	5.2 (3.5,7.8)
Trastuzumab N297G+S1	5.3 (4.5,6.1)
Trastuzumab N297G+S4	5.3 (4.5,6.3)
Trastuzumab N297G+S6	5.7 (4.7,6.8)
Trastuzumab N297G+S146	6.4 (5.8,7)

Table S3: Equilibrium dissociation constant values (K_D) for antibody/FcRn Binding

**Antibody Construct**	**K_D (nM) (95% CI) for FcRn Binding**
F5111 WT	107.4 (50.5,233)
F5111 N297A	132.5 (41.8,449.5)
F5111 N297A+S1	104.7 (40.4,278.8)
F5111 N297A+S2	ND
F5111 N297A+S3	398.7 (189.9,1019)
F5111 N297A+S4	174.7 (63.7,521.1)
F5111 N297A+S5	101.9 (47.5,222.5)
F5111 N297A+S6	116.4 (44.7,312.3)
F5111 N297A+S14	53 (27.2,107.1)
F5111 N297A+S34	142.4 (36.2,881)
F5111 N297A+S146	64 (35.6,117.7)
F5111 N297A+S346	129.6 (21.7,1936)
Trastuzumab N297G	43.2 (30,62.4)
Trastuzumab N297G+YTE+SEFL	20.4 (13,32.1)
Trastuzumab N297G+S146	41.4 (17.2,103.7)
Trastuzumab N297G+YTE+SEFL+S146	39.2 (23.9,64.9)

Table S4: Equilibrium dissociation constant values (K_D) for antibody/FcγRI binding

**Antibody Construct**	**K_D (nM) (95% CI) for FcγRI**
F5111 WT	8.5 (5.7,12.7)
F5111 N297A	105.1 (62.3,182.6)
F5111 WT+S1	9.3 (6.4,13.6)
F5111 WT+S2	8.9 (6.4,12.4)
F5111 WT+S3	7.5 (5.6,10.1)
F5111 WT+S4	14 (6.6,30.4)
F5111 WT+S5	4.8 (3.5,6.8)
F5111 WT+S6	6.5 (4.8,8.8)
F5111 N297A+S14	9.3 (7.9,11.1)
F5111 N297A+S34	9.9 (6.9,14.3)
F5111 N297A+S146	10.9 (6.6,18.2)
F5111 N297A+S346	13.4 (7.2,25)
F5111 N297A+S14	9.3 (7.9,11.1)

Table S5: Half maximal inhibitory concentrations (IC_{50}) for trastuzumab glycovariant ADCs against $HER2^+$ cell lines.

Construct	IC_{50} SKBR3 (nM) (95% CI)	IC_{50} MDA-MB-453 (nM) (95% CI)	IC_{50} HCC1954 (nM) (95% CI)
Trastuzumab N297G	n.d.	0.23 (0.13,0.39)	n.d.
Trastuzumab N297G+S1	0.79 (0.6,1.1)	0.13 (0.11,0.16)	6.1 (1.2,31)
Trastuzumab N297G+S4	0.65 (0.44,0.96)	0.24 (0.15,0.38)	2.0 (0.35,20)
Trastuzumab N297G+S6	1.7 (0.92,3.3)	0.11 (0.087,0.14)	4.4 (0.11,32)
Trastuzumab N297G+S146	0.17 (0.12,0.24)	N.A.	0.45 (0.3,0.68)
DBCO-PEG4-Val-Cit-PAB-MMAE	98 (63,150)	89 (48,160)	131 (74,240)

Table S6: Melting temperatures for trastuzumab glycovariants and their respective conjugates.

Construct	Tm1	Tm2
Trastuzumab N297G	61.69	80.26
Trastuzumab N297G+MMAE	62.03	80.59
Trastuzumab N297G+S1	55.94	80.26
Trastuzumab N297G+S1+MMAE	55.26	79.92
Trastuzumab N297G+S4	61.02	81.27
Trastuzumab N297G+S4+MMAE	61.02	81.94
Trastuzumab N297G+S6	61.69	83.62
Trastuzumab N297G+S6+MMAE	62.03	81.16
Trastuzumab N297G+S146	55.74	82.96
Trastuzumab N297G+S146+MMAE	54.57	82.29
Trastuzumab N297G+YTE+SEFL	73.21	81.59
Trastuzumab N297G+YTE+SEFL+S146	60.41	82.94
